# Surveillance of enteropathogenic Escherichia coli in England, 2011–2024

**DOI:** 10.1099/jmm.0.002208

**Published:** 2026-09-18

**Authors:** Jennifer N. Christopher, Ella V. Rodwell, Satheesh Nair, Marie Anne Chattaway, Claire Jenkins

**Affiliations:** 1 Gastrointestinal Bacteria Reference Unit, UK Health Security Agency, London NW9 5HT, UK; 2 Gastrointestinal Infections, Food Safety & One Health Division, UK Health Security Agency, London NW9 5HT, UK; 3 National Institute for Health and Care Research Health Protection Research Unit in Gastrointestinal Infections, University of East Anglia, Norwich NR4 7TJ, UK

**Keywords:** antimicrobial resistance, enteropathogenic *Escherichia coli*, genome sequencing, surveillance, travellers’ diarrhoea

## Abstract

*An erratum of this article has been published full details can be found at* *https://doi.org/10.1099/jmm.0.002214*

**Introduction.** Enteropathogenic *Escherichia coli* (EPEC) is a diarrhoeagenic pathotype of *E. coli*, historically associated with gastrointestinal (GI) disease in infants and young children.

**Hypothesis/Gap statement.** Although EPEC was the subject of extensive microbiological and epidemiological investigations throughout the twentieth century, in recent years, surveillance of diarrhoeagenic *E. coli* (DEC) has focused on Shiga toxin-producing *E. coli*. Consequently, the true clinical and community burden of EPEC is unknown.

**Aim.** The aim of the study was to review the microbiological typing data and demographic data linked to EPEC isolates and cases diagnosed from 2011 to 2024 in England and to assess the risk to public health.

**Methodology.** Faecal samples that tested positive by PCR for DEC at local microbiology diagnostic laboratories were referred to the UK Health Security Agency (UKHSA) for confirmation and culture. Isolates identified as EPEC were sequenced on the Illumina HiSeq and NextSeq platforms. Sequence type, serotype and antimicrobial resistance (AMR) profile were derived from the genome sequence. Age, sex and travel histories were linked to the typing data.

**Results.** A total of 3,091 EPEC isolates from cases resident in England were notified to the UKHSA between January 2011 and October 2024, with 96% classified as atypical EPEC (aEPEC). The number of referred isolates increased more than eightfold, from 78 in 2011 to 654 in 2024, likely due to the widespread implementation of PCR for the detection of GI pathogens at the local hospital level. There was a slightly higher proportion of females (51.7%), and the highest proportion of cases occurred in the 0–4 age group (30.1%). Reported symptoms included diarrhoea (86.57%) and/or bloody diarrhoea (24.25%) and abdominal pain (9.05%), and 33 cases were diagnosed clinically with haemolytic uraemic syndrome. The population structure was diverse with 447 different serotypes identified, and 24.5% of isolates were resistant to three or more antibiotic classes.

**Conclusion.** A high proportion of cases were infants or young children. Strains causing travellers’ diarrhoea exhibited multidrug resistance and extensive drug resistance. Further research on potential animal reservoirs, likely food vehicles, exposure risks and pathogenic potential of EPEC, is warranted. To facilitate future investigations, we recommend that EPEC should be part of the diagnostic algorithm in the UK and that surveillance strategies should include analysis of travel data and monitoring AMR to inform the global perspective.

## Introduction

Enteropathogenic *Escherichia coli* (EPEC) is a diarrhoeagenic pathotype of *E. coli*, historically associated with gastrointestinal disease in infants and young children [1]. First described as a causative agent of infantile diarrhoea in the 1940s [2], EPEC was the subject of extensive microbiological and epidemiological investigation throughout the twentieth century [3, 4]. It is particularly known for its ability to cause attaching and effacing lesions on the intestinal mucosa, a characteristic feature that differentiates it from commensal strains of *E. coli* [5, 6]. EPEC attaches to intestinal epithelial cells, causing effacement of the microvilli, which in turn leads to a reduced absorption of the surface area and subsequent mild but profuse and persistent diarrhoea. This process is orchestrated by a type III secretion system encoded on the locus of enterocyte effacement (LEE) [3, 4, 7]. EPEC strains are categorized into two subgroups: typical EPEC (tEPEC) and atypical EPEC (aEPEC). tEPEC carries both LEE and the EPEC adherence factor (EAF) plasmid, which encodes the bundle-forming pilus (bfp), essential for localized adherence [8]. In contrast, although aEPEC carries the LEE pathogenicity, it lacks the EAF plasmid and does not produce bfp [8, 9].

The primary mode of EPEC transmission is faecal–oral, typically through contaminated hands, surfaces, food or water [10]. Preventive strategies focus on hygiene, especially in childcare environments. While EPEC infection is generally self-limiting in healthy individuals, it can cause severe dehydration and complications in young children, the elderly or immunocompromised individuals [11]. Supportive care such as oral rehydration remains the most effective way of management. Antibiotic treatment is rarely required; however, it may be required in persistent infections [9].

Although the EPEC pathotype is recognized as a globally significant bacterial pathogen that causes diarrhoeal disease, particularly in infants and young children in developing countries, there is debate as to whether or not it is a true gastrointestinal pathogen [11–16]. EPEC are frequently isolated from both symptomatic and asymptomatic human cases and are ubiquitous in the animal reservoir, food and the environment [15, 17–20]. Certain studies propose that strains belonging to the EPEC group are part of the commensal gut flora and contribute to the intensification of symptoms only when other gastrointestinal pathogens are present and/or that pathogenicity is dependent on the presence of additional virulence factors [15, 21]. However, the association of EPEC with both infantile diarrhoea and travellers’ diarrhoea suggests that this pathotype does have a role to play in the disease process [22, 23].

The LEE pathogenicity island (PAI) is also found in certain serotypes of Shiga toxin-producing *E. coli* (STEC) that emerged as a major public health concern in the 1980s, most notably STEC O157:H7 and STEC O26:H11. Studies showed that these highly pathogenic STEC serotypes emerged from EPEC progenitor strains following the acquisition of bacteriophage encoding the Shiga toxin gene [24, 25]. These STEC/EPEC hybrid strains cause severe clinical outcomes, including bloody diarrhoea and haemolytic uraemic syndrome (HUS), a systemic condition characterized by acute renal injury, thrombocytopenia and microangiopathic haemolytic anaemia, that can be fatal. Consequently, in recent decades, clinical and public health research on EPEC has declined, and surveillance strategies have focused on STEC.

Although the focus on STEC is justified, there are reasons for maintaining surveillance of EPEC, not least because our understanding of its pathogenic potential and how that relates to the association with childhood diarrhoea and travellers’ diarrhoea is limited. Furthermore, EPEC have been associated with multidrug resistance (MDR) [22, 26, 27]. We conducted a retrospective analysis of genome sequences of EPEC isolated from faecal specimens referred to the Gastrointestinal Bacteria Reference Unit (GBRU) at the UK Health Security Agency (UKHSA) between 2011 and 2024 to look for epidemiological trends, determine the population structure and identify virulence and antimicrobial resistance (AMR) profiles. By integrating patient demographics and genomic findings, this study aims to present an overview of the passive surveillance data to assess the risk to public health impact of EPEC in England.

## Methods

### Microbiology

Clinical infection is diagnosed by detecting EPEC in faecal specimens using PCR targeting *eae*. Although EPEC are not included in the local hospital diagnostic microbiology testing algorithm in the UK, faecal specimens submitted to the GBRU for testing for STEC are concurrently tested for EPEC [28]. The STEC PCR used at the GBRU targets *stx1*, *stx2* and *eae*. Isolates that test positive for *stx1* and/or *stx2* with or without *eae* are defined as STEC. Isolates that test negative for *stx* and positive for *eae* are defined as EPEC.

All faecal specimens submitted to GBRU were inoculated onto MacConkey agar and into tryptone soya broth (TSB) and incubated at 37 °C. Following overnight incubation, DNA was extracted from TSB using InstaGene (Bio-Rad Catalogue No. 732-6030) and tested using the EU-RL VTEC/EPEC real-time PCR primers and probes detecting *stx* and *eae* on a Rotor-Gene Q (Qiagen, UK) as described previously [28]. For all faecal specimens that tested positive for *stx* and/or *eae*, ten colonies were selected from the MacConkey agar and retested using the same PCR [28].

Prior to 2016, *E. coli* isolates were phenotypically serotyped by the agglutination of antibodies raised in rabbits to the LPS O antigen [29]. From 2011 to 2015, H-typing was not performed at GBRU, and so only serogroup data was available for those EPEC isolated during that time frame. From 2016, all *E. coli* isolates were sequenced, and O and H antigens were derived from the genome data, as described previously [30]. Microbiological results are stored in the Gastro Data Warehouse (GDW), an in-house UKHSA database for storing and linking patient demographic and microbiological typing data.

### DNA extraction, genome sequencing and data quality control

Genomic DNA from EPEC isolated from 2016 to 2024 was extracted using the QIAsymphony (Qiagen). The sequence library was prepared using the Nextera XT DNA and Nextera Flex sample preparation kits (Illumina) and sequenced using the Illumina HiSeq 2500 and NextSeq 1000 platforms (100 bp paired-end reads) at UKHSA. Trimmomatic (v0.32) was used to trim sequence adapters. Reads were discarded when the PHRED score was <30 from the leading and trailing ends, or the read length was <50 bp. FASTQ reads from all sequences in this study can be found at the UKHSA Pathogens BioProject at the National Center for Biotechnology Information (BioProject number PRJNA315192).

### Sequence typing

Sequence type (ST) assignment was performed using a modified version of short-read sequence typing (SRST) using the multilocus sequence type (MLST) (v2.15-2.17) database described by Tewolde *et al*. [31]. The MOST software (for MLST) is available at https://github.com/phe-bioinformatics/MOST. GrapeTree was employed to generate a minimum spanning tree using the Achtman 7 Gene MLST (MSTree-V2) [32]. This was visualized in the GrapeTree platform and annotated with ST derived from SRST2. Probable or Partial STs, due to the database GeneFinder (v2.9–2.11) not holding all the allelic variants, were labelled with ‘P’ in Table S1, (available in the online Supplementary Material). All STs were checked in EnteroBase and were labelled Novel if the profile could not be matched to an established ST [33].

### Virulence and AMR profiling

Detection of *eae* and *bfpA* genes and AMR determinants was performed using GeneFinder (v2.9–2.11) (https://github.com/phe-bioinformatics/gene_finder) [34]. Genes were confirmed to be present if the coverage and sequence similarity were over 85% compared to the reference gene.

Isolates were classified according to the number of antimicrobial classes with detected AMR determinants in whole-genome sequencing data: susceptible (0 classes), resistant (1–2 classes), multidrug-resistant (3–5 classes) and extensively drug-resistant (XDR; >5 classes).

### Epidemiological data

Patient information, including sex, age, clinical presentation and recent travel (less than 7 days prior to onset of symptoms), was collected from laboratory request forms upon submission and stored in GDW. Information on clinical presentation and recent travel history is poorly recorded by general practitioners on the laboratory submission forms.

### Case definition

A confirmed case of EPEC was defined as a faecal specimen from a clinical or asymptomatic case, testing (i) PCR and whole genome sequencing (WGS) positive for *eae* gene and negative for *stx* genes confirmed by GBRU or (ii) culture positive for EPEC. Cases had a sample date between January 2011 and October 2024 for PCR testing and between January 2016 and October 2024 for WGS testing. The date of receipt at GBRU was used to determine if the case was in the study time frame.

## Results

### Overview of demographic data

A total of 3,091 EPEC human isolates were notified to the UKHSA between January 2011 and October 2024 (Table S1). The number of referred isolates increased more than eightfold, from 78 in 2011 to 654 in 2024. Between 2011 and 2016, the number of cases fluctuated between 39 and 82, remaining relatively low. There was a year-on-year increase between 2017 and 2019, and notifications decreased in 2020, before increasing again from 2021 to 2024 (Fig. 1a). A clear seasonal trend was observed, with higher case numbers reported during the summer season, June to September (Fig. 1b).

**Fig. 1.**
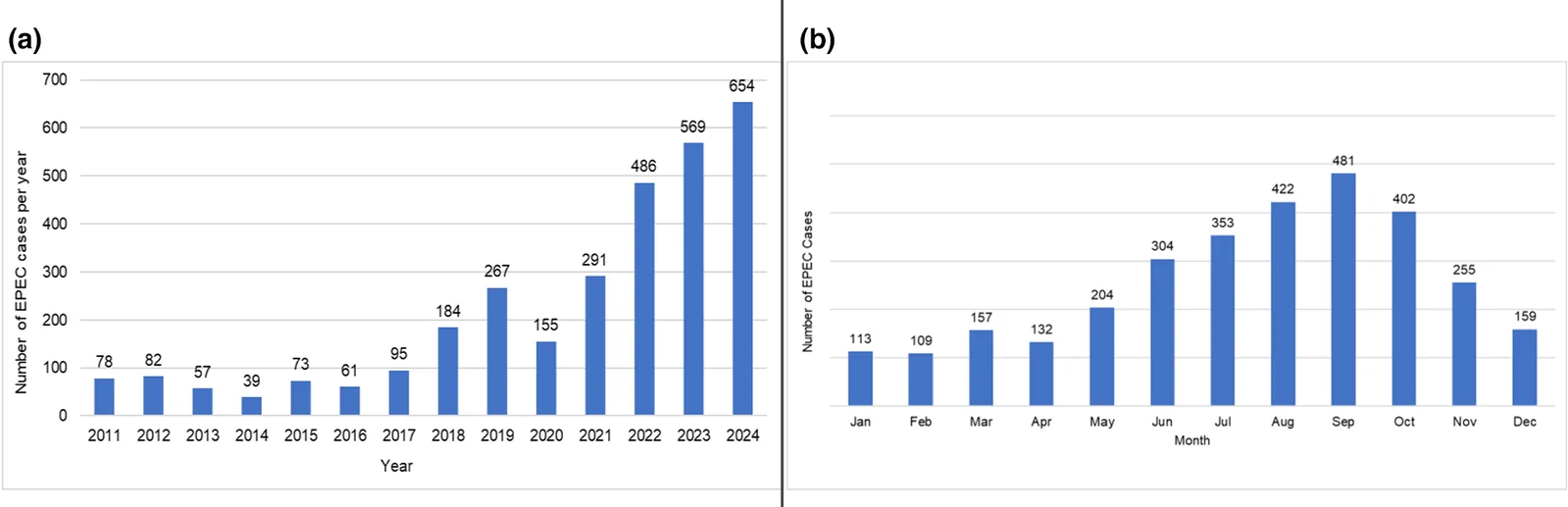
Trends and seasonality of EPEC cases in England in 2011–2024. (**a**) Annual incidence of EPEC reported by GBRU, UKHSA. (**b**) Seasonal trend of the average number of EPEC cases.

For cases where age and sex data were available (*n*=3,057/3,091), 48.28% (1,476/3,057) were male and 51.72% (1,581/3,057) were female. There was a higher proportion of females in the adult age groups and a higher proportion of males in cases aged 16 and younger (Fig. 2). The highest proportion of cases occurred in the 0–4 age group (920/3,057; 30.09%). Within this age group, the proportion of male cases (515/3,057; 16.85%) was higher than that of females (405/3,057; 13.25%), with a male-to-female ratio of 1.27:1 (Fig. 2).

**Fig. 2.**
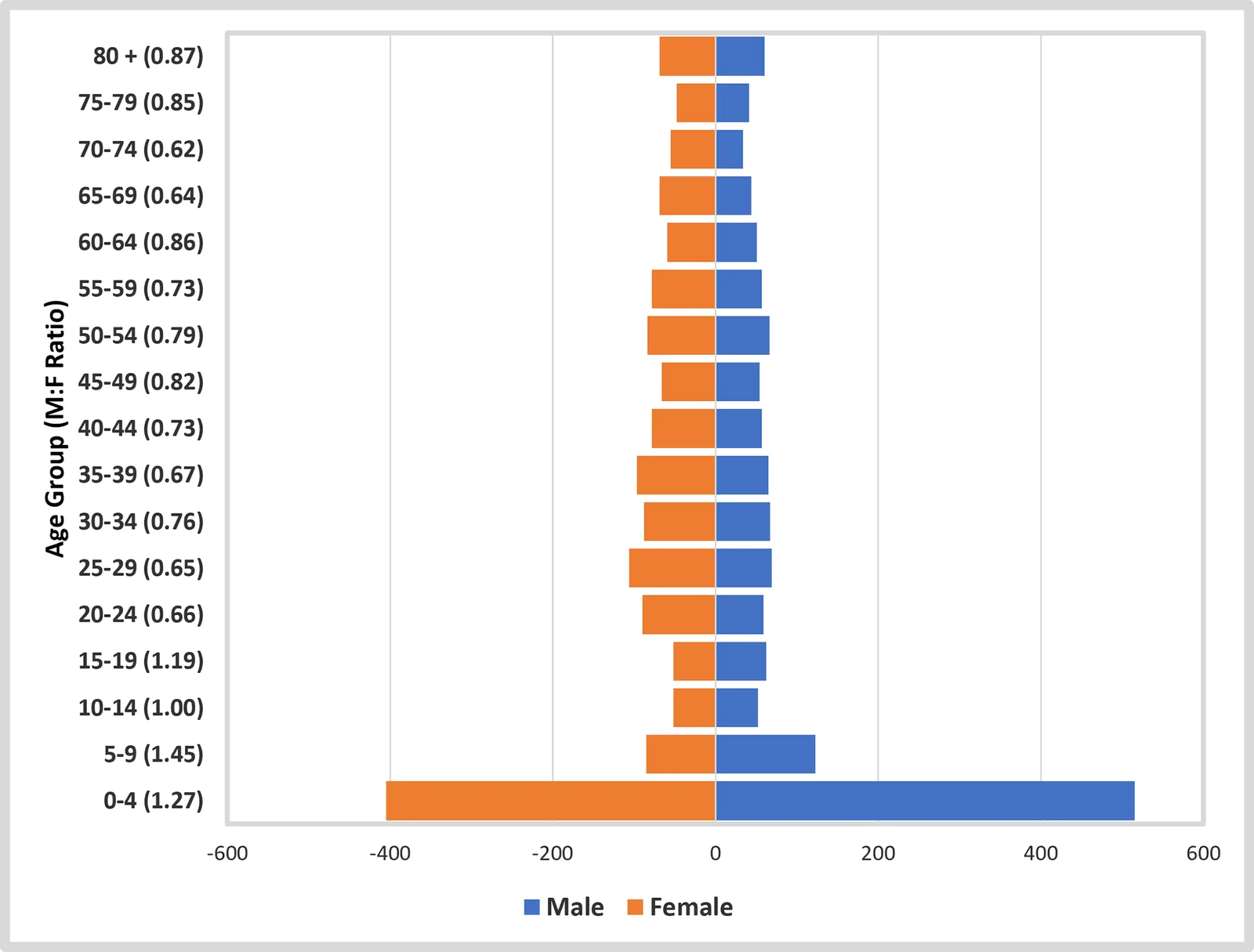
Age–sex distribution of EPEC cases in England (*n*=3,057). Only isolates with receipt date, date of birth, age and sex information were included. Orange, female; blue, male.

Geographical distribution of EPEC cases in England by regions was stated in 2,677 cases. The highest number of EPEC cases was in London (484/2,677; 18.08%), followed by Southeast (467/2,677; 17.44%) and Northwest (449/2,677; 16.77%). East of England and Northeast regions had the lowest cases by 5.86% (157/2,677) and 6.13% (164/2,677), respectively (Table 1). When adjusted for population size, the highest incidence rates were observed in the Northeast (5.94 cases per 100,000 population), followed by the Northwest (5.80 per 100,000), London (5.53 per 100,000) and Yorkshire and the Humber (5.20 per 100,000). The Southeast demonstrated a moderate incidence rate of 4.84 per 100,000 population despite reporting a high number of cases. The lowest incidence rate was observed in the East of England (2.38 per 100,000 population), followed by the Southwest (3.60 per 100,000 population) (Table 1).

**Table 1.** The number of cases and incidence per 100,000 population in each UKHSA region from 2011 to 2024

Region	Population	No. of EPEC isolates	Incidence per 100,000
Southeast	9.64 million	467	4.84
London	9.09 million	484	5.53
Northwest	7.74 million	449	5.80
East of England	6.58 million	157	2.38
West Midlands	6.19 million	239	3.86
Southwest	5.89 million	212	3.60
Yorkshire and the Humber	5.67 million	295	5.20
East Midlands	5.06 million	196	3.87
Northeast	2.76 million	164	5.94

Of the 3,091 EPEC cases included in this study, 486 (15.72%) reported travel outside the UK to 68 different countries. Travel data is poorly captured on the laboratory submission forms, and the absence of a travel history does not mean the case did not travel. The most common international travel destinations were Pakistan (*n*=65), India (*n*=63), Egypt (*n*=61), Turkey (*n*=36), Mexico (*n*=25) and Spain (*n*=22). Travel to each of the remaining 62 countries was reported in fewer than 20 cases (Tables S1 and S2).

### Clinical presentation

Clinical outcome data was available for 1,802/3,091 (58.30%) patients. The most reported symptom across all age groups was diarrhoea (1,560/1,802; 86.57%), including bloody diarrhoea (437/1,802; 24.25%) and watery diarrhoea (61/1,802; 3.39%). Abdominal pain, nausea and pyrexia were reported in 163/1,802 (9.05%), 35/1,802 (1.94%) and 9/1,802 (0.50%) of cases, respectively (Table 2). There were 33/1,802 (1.83%) patients diagnosed clinically with HUS, a systemic condition characterized by microangiopathic haemolytic anaemia (red blood cell destruction), thrombocytopenia (low platelets) and acute kidney injury (kidney failure) (Tables 2 and S3). During a similar time frame, there were 626 patients diagnosed with STEC-HUS [35]. Nearly half of HUS cases occurred in children under 5 years of age (14/33; 42.42%), and two fatal cases were recorded in the 0–4 age group (Table S3).

**Table 2.** Clinical presentation of EPEC in this dataset where clinical presentation data was available (*n*=1802)

Age group	Abdominal pain	Diarrhoea	Bloody diarrhoea	Watery diarrhoea	Nausea	Pyrexia	HUS	Fatal	Total
**0–4**	26	303	128	13		12	14	2	498
**5–9**	15	60	33	1		1	4		114
**10–14**	14	29	19	2	1	4	3		72
**15–19**	8	39	18	2	2	3	4		76
**20–24**	7	46	33	3	1		2		92
**25–29**	14	58	37	5		3	1		118
**30–34**	14	48	24	3		2			91
**35–39**	10	61	21	4	1	2			99
**40–44**	12	45	19	4	1	3	1		85
**45–49**	7	41	15	3	1	1			68
**50–54**	8	49	20	5		1	1		84
**55–59**	8	63	10	2	1				84
**60–64**	5	43	13	2					63
**65–69**	7	53	8	3			1		72
**70–74**	3	37	11	3			1		55
**75–79**	3	34	15	2		1			55
**80–84**		27	6	1		2			36
**85–89**	2	19	5				1		27
**90–94**		5	1	3	1				10
**95–99**		1	1						2
**100–104**		1							1
**Total**	163	1,062	437	61	9	35	33	2	1,802

### Serotype, ST profile and presence of *bfpA*

There were 447 different serotypes identified among the EPEC in this dataset. The three most common Clonal Complexes (CCs) were CC381, CC29 and CC10. All CCs were characterized by high levels of diversity and comprise a wide range of different serotypes (Tables 3 and S1). The most common *E. coli* serogroups identified were *E. coli* O157 (273/3,085, 8.9%), *E. coli* O26 (133/3,085, 4.3%) and *E. coli* O145 (100/3,085, 3.24%). Within each serogroup, the majority of isolates belonged to a limited number of STs, specifically *E. coli* O157, ST11 (*n*=155/273), *E. coli* O26 ST21 or ST29 (*n*=94) and *E. coli* O145 ST137 (*n*=67/100) (Tables 3 and S1). The 33 EPEC (tEPEC *n*=1; aEPEC *n*=32) isolates linked to patients diagnosed with HUS, belonged to over 20 different serogroups, with serotype O26:H11 being the most common (Table S3). The *bfpA* gene was detected in 120/3,091 (3.9%) of the EPEC isolates in this study, incorporating at least 33 different serogroups (Table S1). The most frequently detected serogroups in the tEPEC subset were *E. coli* O157 (ST10 or ST725; *n*=13) and *E. coli* O55 (ST40 or ST5342; *n*=11).

**Table 3.** Serotype and ST diversity of EPEC in this dataset Only serotypes with more than 23 isolates and STs within those serotypes are included. Serotypes with less than 23 isolates are classified as other serotypes. Flagellar typing of 177 isolates of *E. coli* O157 is not stated due to unavailable data (*n*=3,091).

Serotype	ST10	ST11	ST17	ST21	ST28	ST29	ST34	ST40	ST137	ST206	ST300	ST328	ST335	ST343	ST378	ST381	ST517	ST583	ST590	ST752	ST1041	Other STs	Total
**O157**	1	64																		1	1	110	177
**O157:H7**	1	91																				4	96
**O157:H39**																					30	19	49
**O157:H16**	39																					0	39
**O26:H11**				32		94																7	133
**O145:H28**									67											4		29	100
**O33:H6**					53																	2	55
**O101:H33**							20								27							1	48
**O177:H11**						33																13	46
**O109:H21**								43												1		1	45
**O unidentifiable:H6**					36													2				7	45
**O unidentifiable**						1					1					1		1	1			40	45
**O182:H25**											32											12	44
**O156:H25**											25			1								17	43
**O88:H25**												18					19					1	38
**O123:H40**	1																			34		1	36
**O148:H9**																34						0	34
**O unidentifiable:H21**								26														6	32
**O49:H10**										24												8	32
**O103:H2**			24											1								4	29
**O55:H7**													25									3	28
**O unidentifiable:H40**	20							1												4		2	27
**O100:H11**																24						1	25
**O63:H6**																		9				15	24
**O71:H40**	8																			14		1	23
**Other serotypes**	120	1	85	0	40	65	4	30	5	12	12	12	5	19	1	138	114	16	24	37	0	1,058	1,798
**Total**	190	156	109	32	129	193	24	100	72	36	70	30	30	21	28	197	133	28	25	95	31	1,362	3,091

### Antimicrobial resistance

Genome-derived AMR data was available for 2,825/3,091 isolates in this study. Of these, genome-derived AMR determinants were detected in 1,087/2,825 (38.48%), and 499/2,825 (17.66%) were defined as MDR, with 147/499 (29.46%) isolated from patients reporting foreign travel (Figs 3, 4 and 5, Table S2). XDR was identified in 259/2,825 (9.16%) of isolates, with 121/259 (46.72%) of the cases associated with foreign travel. The most common destinations were Egypt (MDR *n*=28/147, 19.05%; XDR *n*=9/121, 7.44%), India (MDR *n*=27/147, 18.37%; XDR *n*=15/121, 12.40%) and Pakistan (MDR *n*=17/147, 11.56%; XDR *n*=33/121; 27.27%) (Fig. 4, Table S2).

**Fig. 3.**
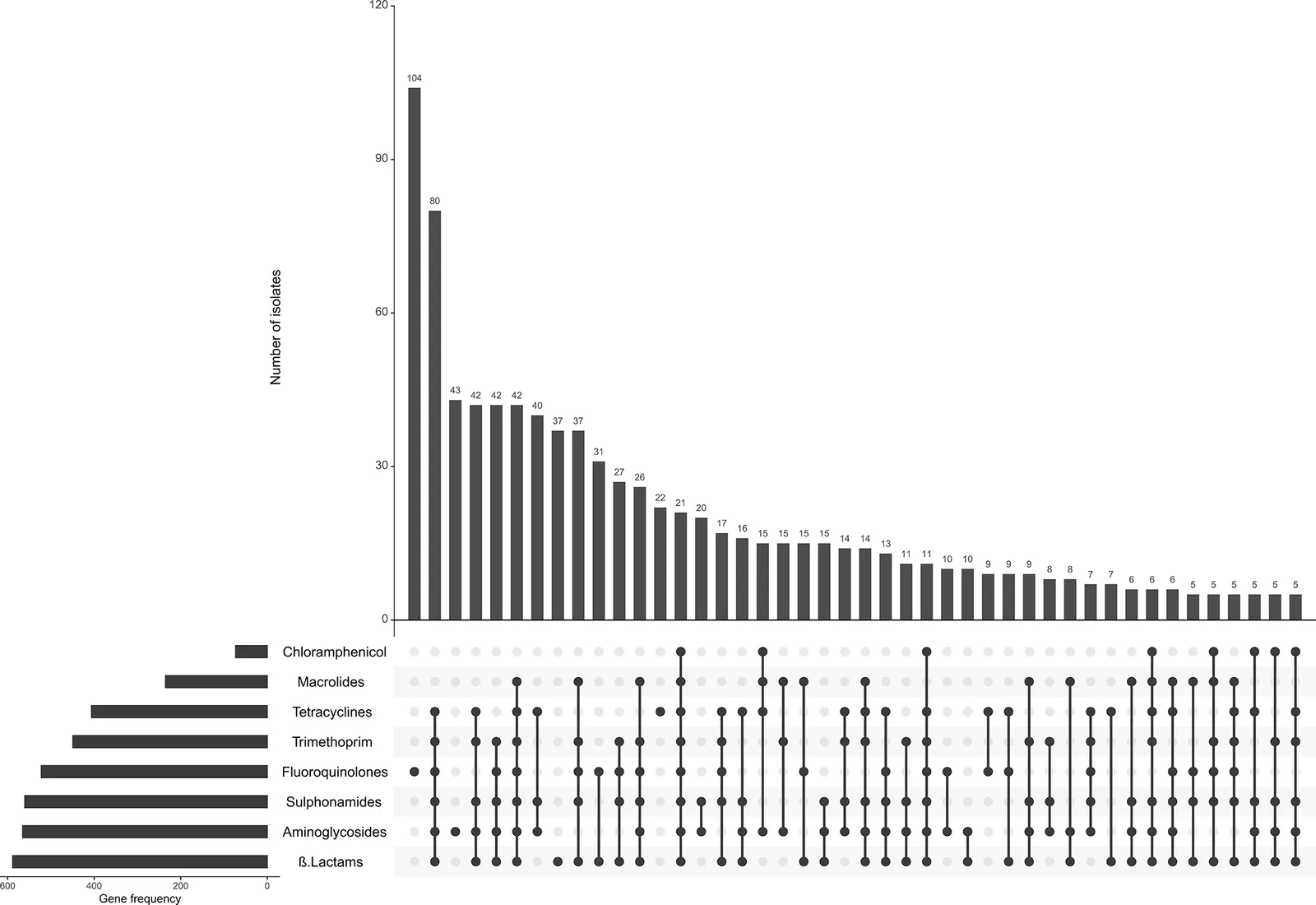
AMR gene class combinations among EPEC isolates in England (*n*=3,091). An UpSet plot showing the frequency and overlap of AMR gene classes detected among EPEC isolates collected in England generated using UpSetR in RStudio. The vertical bars (top panel) represent the number of isolates carrying each unique combination of AMR gene classes. The dot matrix (lower panel) indicates the specific AMR gene classes present in each combination, with filled dots connected by vertical lines denoting co-occurrence within the same isolates. The horizontal bars (left panel) summarize the overall frequency of each AMR gene class across the dataset, including *β*-lactams, aminoglycosides, sulphonamides, fluoroquinolones, trimethoprim, tetracyclines, macrolides and chloramphenicol resistance genes.

**Fig. 4.**
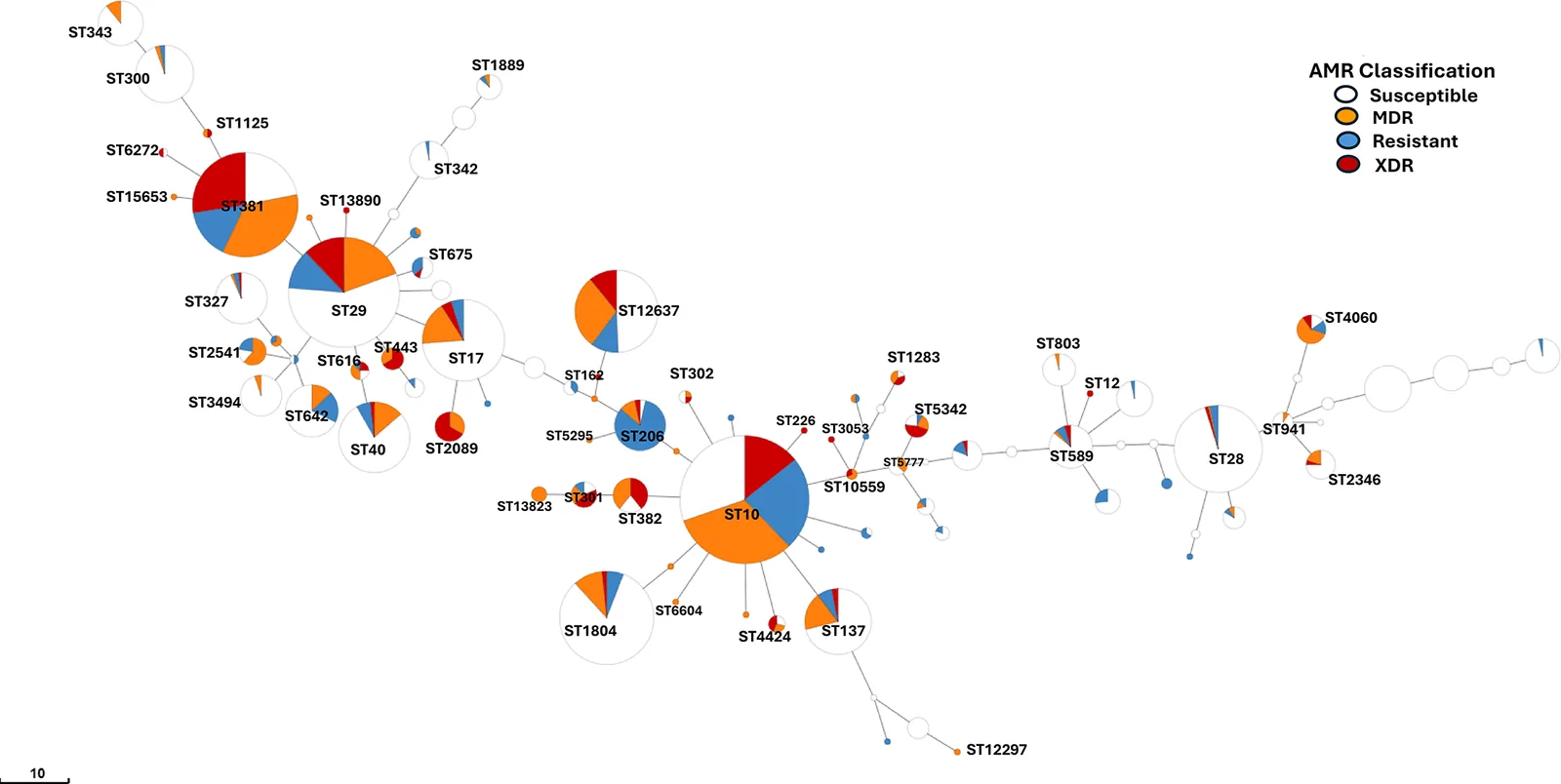
Seven-gene MLST minimum spanning tree showing STs categorized based on AMR classification – susceptible, resistant, MDR or XDR (*n*=2,804). Each node represents an ST, the node size corresponds to the number of isolates, while the colour segments within each node represent AMR profiles categorized by the number of drug classes to which the isolates show resistance. The edges represent allelic differences in the MLST scheme, with the scale bar (10) indicating a maximum of ten allelic differences.

**Fig. 5.**
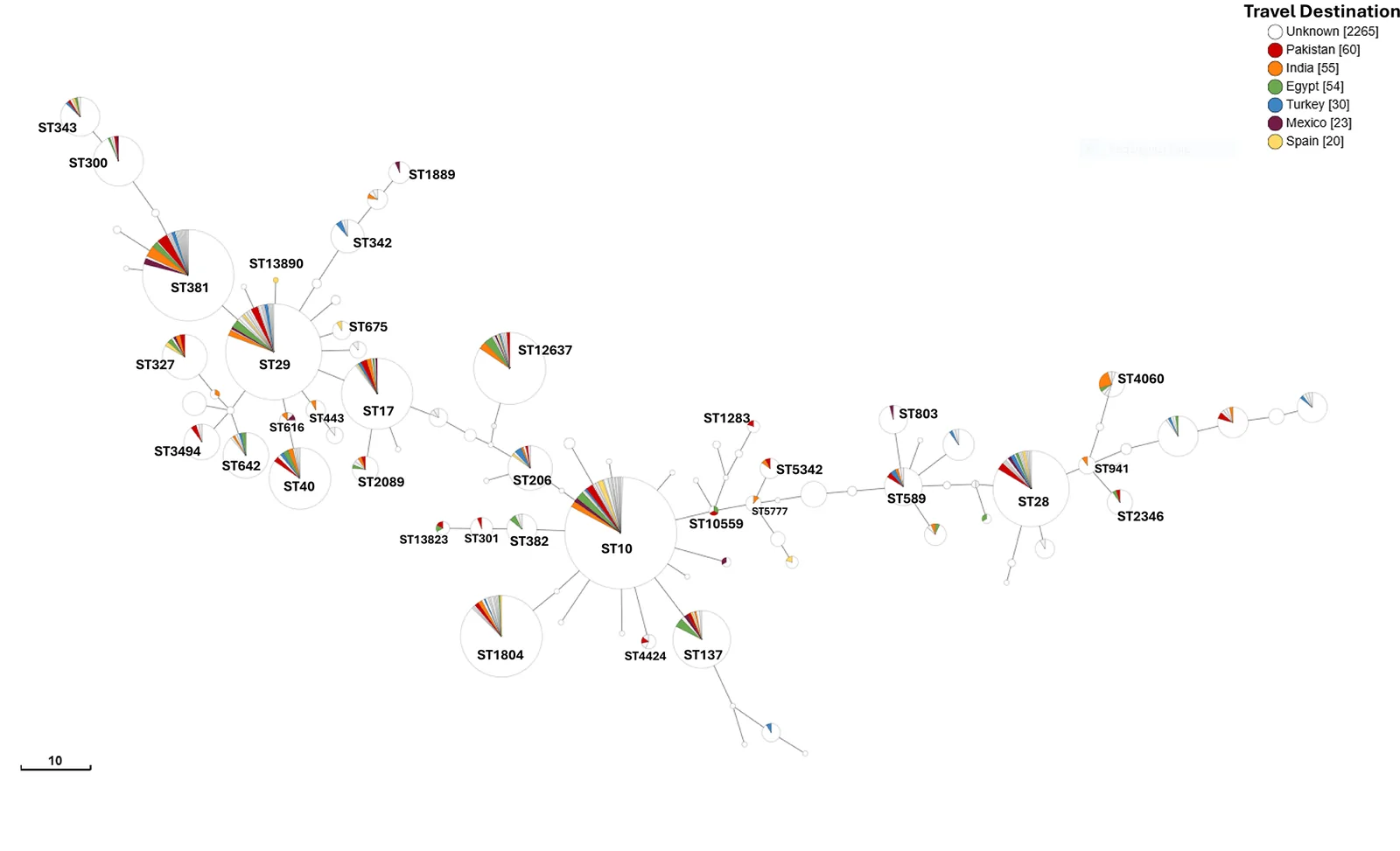
Seven-gene MLST minimum spanning tree showing STs categorized by reported travel destinations (*n*=2,804). Each node represents an ST, the node size corresponds to the number of isolates, while the colour segments within each node represent reported travel destination. The edges represent allelic differences in the MLST scheme, with the scale bar (10) indicating a maximum of ten allelic differences.

The highest proportion of isolates had AMR gene determinants known to confer resistance to the *β*-lactam class of antibiotics (696/2,825; 24.63%), with the most common determinants being *bla_TEM-1_* (462/696; 66.38%) and *bla_CTX-M-15_* (205/696; 29.45%). There were 637/2,825 (22.55%) of isolates that had AMR gene determinants conferring reduced susceptibility to the fluoroquinolones, *qnrS-1* (301/637; 47.25%) or the point mutation in *gyrA*83:S-L (192/637; 30.14%) (Table S1).

AMR genes known to confer resistance to the aminoglycosides were identified in 681/2,825 (22.03%) isolates that had *aph(6)-Id* (518/681;76.06%), and 675/2,825 (23.89%) had AMR gene determinants known to confer resistance to sulphonamides with *sul-1*, *sul-2* or *sul-3* determinants, either individually or in combination. AMR determinants known to confer resistance to trimethoprim (539/3,091), tetracycline (501/3,091), macrolides (319/3,091) and chloramphenicol (113/3,091; 3.66%) were also detected, with *dfrA* gene variants (mainly *dfrA*14, *dfrA*1 and *dfrA*17), *tetA*, *mphA* and *catA*1, respectively, being the most common AMR determinant detected in each class (Table S1).

## Discussion

During the time frame of this study, notifications of EPEC were consistently below 95 cases each year until 2018 when the rate of detection doubled. The eightfold increase in notifications between 2019 and 2024 was most likely due to the widespread implementation of PCR-based assays to detect a wider range of gastrointestinal pathogens at the frontline hospital diagnostic laboratories [36, 37, 38]. In response to the COVID-19 pandemic, equipment, reagents, consumables and trained staff were provided to increase the capacity for molecular testing of respiratory specimens. In certain settings, once the pandemic had abated, these resources were used to improve the diagnostic algorithms for testing faecal specimens from hospitalized patients and community cases of diarrhoea [39]. DEC, including EPEC, are challenging to differentiate from commensal gut *E. coli* using culture alone, and PCR targeting specific virulence genes is required to facilitate detection [22, 28]. Consequently, the increase in EPEC diagnoses was due, at least in part, to improved sensitivity and broader application of molecular testing, and not solely due to higher rates of infection and/or transmission.

Although improvements to the diagnostic algorithms were the most likely explanation for the increasing number of notifications, other factors were considered. In young children, contact with other children and environmental exposures, such as contact with animals in rural settings or swimming in coastal waters, may contribute to building immunity to bacterial pathogens. COVID-19 pandemic lockdown measures, including enhanced hygiene practices, reduced social interaction, the closure of schools and nursery schools and limited exposure to the natural environment, may have contributed to creating an immunity gap [40, 41]. Although robust scientific research on this topic is sparse, and the evidence base is not well established, it has been suggested that disrupting the development of a healthy gut microbiome may contribute to children being more vulnerable to infection with gastrointestinal pathogens, such as EPEC, in the post-pandemic era [40, 42].

EPEC are considered a cause of paediatric diarrhoeal disease in low- and middle-income countries, and this study provides evidence that EPEC continues to contribute to a high burden of paediatric diarrhoea in England [11, 15, 16, 23]. In line with trends observed with other diarrhoeagenic *E. coli* pathotypes, the under 5 years age group showed a high proportion of cases [38, 43]. Symptomatic EPEC cases demonstrated a clear age-dependent pattern, with young children disproportionately affected across a range of clinical presentations, including diarrhoea, abdominal pain, pyrexia and HUS. These findings reflect the well-established vulnerability of infants and toddlers to EPEC infection, likely due to immature immune systems and poor hygiene behaviours [9]. Smaller secondary peaks were observed in young adult and middle-aged groups, and factors such as recreational travel and evolving dietary habits in this demographic may contribute to distinctive exposure patterns [44]. Case counts declined progressively with increasing age, particularly in those over 65 years.

The inclusion of cases diagnosed with HUS and/or bloody diarrhoea in this study and elsewhere has raised questions as to whether EPEC alone can cause these symptoms [45–49]. The predominance of HUS cases in the under 5 years age group is consistent with previous findings that young children are particularly susceptible to severe outcomes of EPEC and other diarrhoeagenic *E. coli* infections [9, 50, 51]. It is plausible that some of HUS cases identified in this study may have resulted from coinfections with STEC, rather than EPEC alone, and that the STEC was undetected or had cleared by the time testing was commenced. Alternatively, these cases may represent examples where the patient was infected with STEC, but the strain of *E. coli* lost the genes encoding Shiga toxin *in vivo* [45, 52].

Phylogenetic analysis of the EPEC pathotype revealed a diverse group belonging to a wide range of different CCs and STs, exhibiting a variety of different serotypes within each ST. The presence of *eae* is the defining characteristic of the group and is located on the LEE PAI. The LEE PAI mobile genetic element appears to have been horizontally dispersed across the *E. coli* population structure and, once acquired, has persisted in an extensive range of STs [8]. The diversity across serotypes, STs and clonal complexes reflects the organism’s adaptive versatility, variety of animal reservoirs for aEPEC and wide range of environmental niches. Notably, *E. coli* O157 accounted for a high proportion of the isolates, providing further evidence that this serogroup has pathogenic potential with and without *stx* [53, 54]. Of the previously described and well-established EPEC serogroups, O26, O55, O86, O111, O114, O119, O125, O126, O127, O128, O142 and O158, only EPEC O26 and O55 were frequently identified in our study [55, 56]. At the clonal complex level, CC10 was the third most common CC after CC381 and CC29. CC10 is known for its broad ecological range and frequent representation among aEPEC strains [57]. Overall, the data indicate that while a small number of serotype–ST lineages dominate, there remains considerable genomic diversity across the collection, consistent with previous findings from genomic epidemiology of diarrhoeagenic *E. coli* [43, 58, 59].

Previous studies have shown that AMR may drive the emergence and persistence of gastrointestinal pathogens circulating in the community [60–63]. Over 30% of isolates in this study were resistant to at least one class of antibiotic, most commonly to the *β*-lactam antibiotics. These trends align with broader findings in *E. coli* surveillance, which have consistently reported high resistance to commonly used oral and first-line antimicrobials [64]. Carbapenem resistance remains rare in EPEC in the UK, likely due to restricted clinical use [65]. Resistance to chloramphenicol was also relatively low, and this preserved susceptibility could reflect its reduced use in clinical practice in recent years, limiting selection pressure [66].

In line with previous studies from UKHSA, only 4% of the isolates observed in this study were tEPEC [23]. tEPEC were mostly responsible for the high mortality associated with infantile diarrhoea in the 1940s and subsequent decades. Despite the high proportion of isolates from children under the age of 5 years old, tEPEC were rare in this dataset. The most common tEPEC serogroups were O157 (serotypes O157:H16 or O157:H45) and O55 (serotypes O55:H21 or O55:H51), both serogroups associated with STEC (O157:H7 and STEC O55:H7), although with different associated H-types.

Approximately 16% of isolates exhibited MDR, and these figures reflect global patterns in increasing AMR among enteric pathogens, particularly those associated with travel-acquired infections [63]. MDR and XDR isolates were identified in multiple STs, including dominant lineages such as ST10, ST29, ST381 and ST28, indicating that AMR is not limited to specific travel-associated or clonal lineages but is instead widespread across the EPEC population circulating in England. Although travel history data is poorly collected by general practitioners and known to be grossly under-reported, a high proportion of the EPEC isolated from travellers were MDR or XDR. The isolates exhibiting MDR and XDR were commonly isolated from travellers returning from regions where antibiotics are widely available, but guidance and stewardship are limited [67–70]. These findings align with growing global evidence that travel to regions with a high prevalence of AMR significantly contributes to the importation and dissemination of resistant *E. coli* strains [71, 72]. Furthermore, there is concern regarding the role of horizontal gene transfer in the dissemination of AMR determinants from AMR *E. coli* pathotypes to extraintestinal *E. coli*, and the risk of treatment failures as antibiotics used for empirical treatment may be rendered ineffective [73, 74] (Antimicrobial Resistance Collaborators).

The third Infectious Intestinal Disease (IID3) study, carried out in the UK from October 2023 to September 2025, identified EPEC as the most commonly detected pathogen among cases of community-acquired diarrhoea (Microsoft Power BI) [75]. EPEC have been isolated from animals and food, as well as having the potential to be transmitted via person-to-person contact, especially in childcare settings [76–78]. A high proportion of infections were associated with infants and young children, and strains causing travellers’ diarrhoea exhibit MDR and XDR. These findings are all justification that further research on the animal reservoir, likely food vehicles, exposure risks and pathogenic potential of EPEC is warranted. To facilitate future investigations, we recommend that EPEC should be part of the diagnostic algorithm in the UK and that surveillance strategies should include analysis of travel data and monitoring AMR to inform the global perspective.

## Supplementary material

10.1099/jmm.0.002208 Supplementary Material 1.

10.1099/jmm.0.002208 Supplementary Material 2.

## Abbreviations

aEPEC: atypical EPEC
AMR: antimicrobial resistance
bfp: bundle-forming pilus
CCs: clonal complexes
DEC: diarrhoeagenic *Escherichia coli*
EAF: EPEC adherence factor
EPEC: enteropathogenic *Escherichia coli*
GBRU: Gastrointestinal Bacteria Reference Unit
GDW: Gastro Data Warehouse
GI: gastrointestinal
HUS: haemolytic uraemic syndrome
LEE: locus of enterocyte effacement
MDR: multidrug resistance
MLST: multilocus sequence type
PAI: pathogenicity island
SRST: short-read sequence typing
ST: sequence type
STEC: Shiga toxin-producing *Escherichia coli*
tEPEC: typical EPEC
TSB: tryptone soya broth
UKHSA: UK Health Security Agency
WGS: whole genome sequencing
XDR: extensively drug-resistant
