# Erratum: Surveillance of enteropathogenic Escherichia coli in England, 2011–2024

**DOI:** 10.1099/jmm.0.002214

**Published:** 2026-10-07

**Authors:** Jennifer N. Christopher, Ella V. Rodwell, Satheesh Nair, Marie Anne Chattaway, Claire Jenkins

**Affiliations:** 1 Gastrointestinal Bacteria Reference Unit, UK Health Security Agency, London NW9 5HT, UK; 2 Gastrointestinal Infections, Food Safety & One Health Division, UK Health Security Agency, London NW9 5HT, UK; 3 National Institute for Health and Care Research Health Protection Research Unit in Gastrointestinal Infections, University of East Anglia, Norwich NR4 7TJ, UK

During the production process panel b of Fig. 1 was mistakenly excluded from the proofs of the article leading to the publication of an incomplete figure. The correct complete figure is presented in this erratum, and the Version of Record has been updated to reflect this correction.

**Fig. 1.**
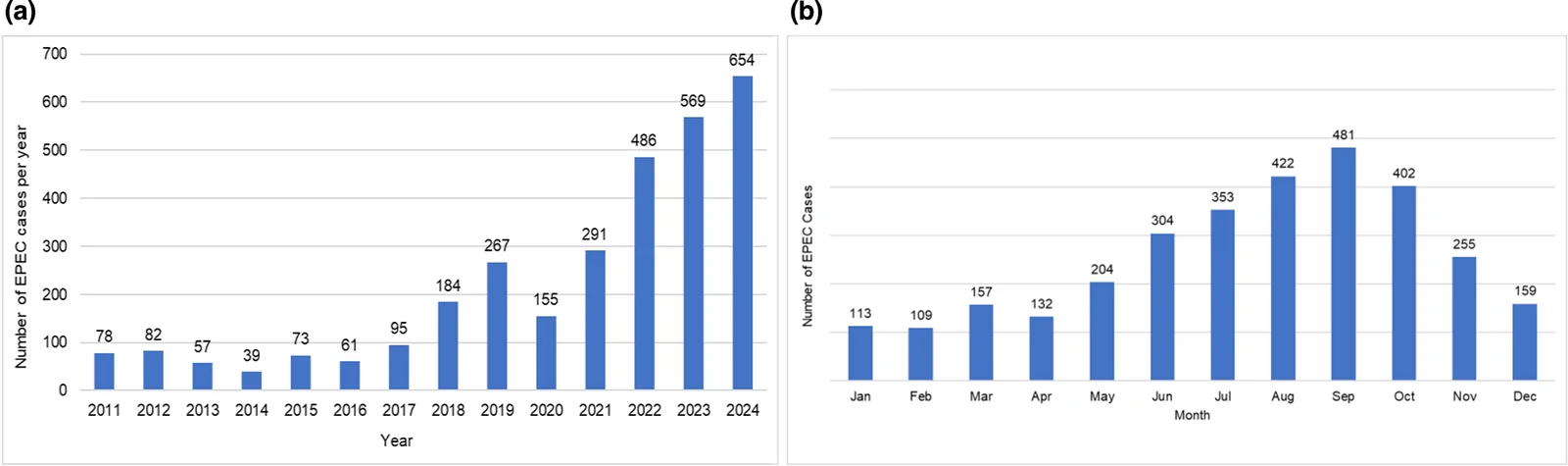
Trends and seasonality of EPEC cases in England in 2011–2024. (a) Annual incidence of EPEC reported by GBRU, UKHSA. (b) Seasonal trend of the average number of EPEC cases.

The Microbiology Society apologizes for any inconvenience this oversight may have caused.

