# Supplementary material for: Surveillance of enteropathogenic Escherichia coli in England, 2011–2024
Source: J Med Microbiol. 2026 Sep 18;75(9):002208. doi: 10.1099/jmm.0.002208 (PMC13645379; doi:10.1099/jmm.0.002208)
Supplement: Supplementary file 1 [file jmm-75-02208-s002.pdf]

Supplementary 2: Distribution of Isolates by Travel Destination and Resistance Category. Resistance categories were defined using genome-derived AMR determinants: susceptible (0 antimicrobial classes), resistant (1–2 classes), MDR (3–5 classes), and XDR (>5 classes).

Travel Destination	Resistant	Susceptible	MDR	XDR	Total
**Pakistan**	7	8	17	33	65
**India**	12	9	27	15	63
**Egypt**	12	12	28	9	61
**Turkey**	7	20	5	4	36
**Mexico**	5	7	5	8	25
**Spain**	2	17	1	2	22
**African continent**	2	5	6	6	19
**Morocco**	3	5	4	3	15
**Cape verde**	2	1	6	5	14
**Germany**		12	1		13
**Asian continent**		4	3	2	9
**European continent**		7	1		8
**Bangladesh**	1	3	3		7
**South africa**	2	1	4		7
**Kenya**		2	1	3	6
**Cuba**	2	2		1	5
**Nepal**		1	2	2	5
**Dominican republic**	1	1	2		4
**Ethiopia**	1	1	2		4
**Ghana**	1		1	2	4
**Romania**		4			4
**Tanzania**	1	1	1	1	4
**Tunisia**	2	3			5
**United Arab Emirates**	1	1	1	1	4
**Afghanistan**		2	1		3
**Bulgaria**	2	1			3
**Colombia**			1	2	3
**Cyprus**		1	1	1	3
**France**		1	2		3
**Gambia**		1	2		3
**Malaysia**		1		2	3
**Middle East (region)**	1		1	1	3
**Peru**	1	1	1		3
**Thailand**		2		2	4
**Caribbean**	2				2
**Italy**		2			2

**Madagascar**		1	1		2
**Portugal**		2			2
**Saudi arabia**			2		2
**Sri lanka**		2			2
**Sudan**		1		1	2
**Tokelau**				2	2
**Uganda**			2		2
**Zimbabwe**			2		2
**Canada**	1				1
**Chile**		1			1
**Denmark**		1			1
**Dominica**			1		1
**Fiji**		1			1
**Greece**		1			1
**Guinea**		1			1
**Indonesia**			1		1
**Iraq**				1	1
**Jordan**				1	1
**Malawi**		1			1
**Namibia**		1			1
**Nigeria**			1		1
**Palestinian territory**				1	1
**Panama**			1		1
**Philippines**		1			1
**Poland**		1			1
**Slovakia**		1			1
**Somalia**		1			1
**Tajikistan**				1	1
**United states**		1			1
**Venezuela**			1		1
**Yemen**				1	1
**Zambia**		1			1
**Unknown Destination**		1	5	8	2
**Total**	71	159	147	121	486

Supplementary 3: Demographic data and microbiological typing data of cases diagnosed with HUS

**SRA Accession**	**Isolation date**	**Age**	**Sex**	**Serotype**	**ST**	**bfpA**	**EAE**
No WGS data	01/11/2011	2	M	O157	n/a	n/a	+

No WGS data	26/03/2012	7	M	O157	n/a	n/a	+
No WGS data	11/09/2012	1	M	O25	n/a	n/a	+
No WGS data	19/08/2013	1	F	O26	n/a	n/a	+
SRR15369481	31/01/2019	16	M	O35:H19	517	-	+
SRR15338016	10/07/2020	86	F	O26:H11	29	-	+
SRR15842436	10/08/2021	1	M	O25:H8	327	-	+
SRR15842425	13/08/2021	1	F	O179:H31	1092	-	+
SRR15842887	13/08/2021	15	F	O179:H31	1092	-	+
SRR17120126	24/11/2021	17	M	O85:H31	803	-	+
SRR17681531	23/12/2021	55	F	O unidentifiable:H6	28	-	+
SRR19333137	06/05/2022	25	F	O119:H8	590	+	+
SRR19431955	19/05/2022	2	F	O114:H49	589	-	+
SRR19973572	23/06/2022	5	M	O132:H34	582	-	+
SRR20210765	05/07/2022	22	F	O126:H34	4101	-	+
SRR21404627	23/08/2022	40	F	O157:H39	4060	-	+
SRR21520321	02/09/2022	70	F	O unidentifiable:H40	40	-	+
SRR21918463	27/09/2022	1	F	O142:H34	2346	-	+
SRR21910662	05/10/2022	1	F	O80:H2	301	-	+
SRR22096633	20/10/2022	26	M	O76:H19	675	-	+
SRR22196939	27/10/2022	65	M	O71:H40	10	-	+
SRR28158826	28/03/2023	5	M	O unidentifiable:H40	10	-	+
SRR29241265	20/06/2023	6	M	O26:H11	21	-	+
SRR29225494	07/07/2023	1	M	O151:H5	206	-	+
SRR29091339	23/10/2023	2	F	O unidentifiable:H10	10	-	+
SRR29091295	23/10/2023	10	F	O10:H25	10988	-	+
SRR29079826	02/11/2023	6	M	O145:H34	713	-	+
SRR29077631	24/11/2023	10	F	O26:H11	21	-	+
SRR27696254	09/01/2024	3	M	O26:H11	21	-	+
SRR28891267	25/04/2024	14	F	O70:H40	10	-	+
SRR29499083	05/06/2024	7	M	O174:H7	2541	-	+
SRR29495075	13/06/2024	3	F	O167:H9	2538	-	+
SRR29672706	24/06/2024	15	M	O unidentifiable:H6	28	-	+
